# Effect of Inadequate Treatment in Adult Patients with Community-Acquired Acute Pyelonephritis Due to Enterobacterales Under Empirical Management with Cefazolin

**DOI:** 10.3390/antibiotics14020197

**Published:** 2025-02-14

**Authors:** Laura Cristina Nocua-Báez, Patricia Reyes, Jorge Alberto Cortes

**Affiliations:** 1 Department of Internal Medicine, School of Medicine, Universidad Nacional de Colombia, Bogotá 111321, Colombia; jacortesl@unal.edu.co; 2 Infectious Diseases Service, Hospital Universitario Nacional, Bogotá 111321, Colombia; 3 Infectious Diseases Service, Clínica Universitaria Colombia, Clinica Colsanitas, Bogotá 111321, Colombia; preyes@colsanitas.com

**Keywords:** acute pyelonephritis, urinary tract infections, antimicrobial agents, antibiotics, cephalosporins, cefazolin, enterobacteriaceae, antimicrobial-prescribing practices, drug resistance, microbial

## Abstract

**Background/Objectives**: First-generation cephalosporins are used in some countries, primarily in Latin America and other low-resource regions, as a first-line or alternative empirical treatment for patients with acute pyelonephritis (AP). This study aimed to evaluate the impact of inappropriate empirical therapy with cefazolin on the clinical outcomes of adult patients with community-acquired AP caused by resistant Enterobacterales, requiring hospitalization in two tertiary hospitals in Bogotá. **Methods**: This retrospective cohort study included hospitalized patients with community-acquired AP caused by Enterobacterales who received initial treatment with cefazolin at two tertiary-level institutions in Colombia (January 2013–2020). Inappropriate treatment was defined as a resistant isolate to cefazolin in the urine culture. Outcomes assessed included hospital stay, hospital mortality, and recurrence. **Results**: A total of 1031 patients were admitted, among whom 218 (21.1%) received inappropriate treatment. The mean length of stay was 4.8 (5.1) days, 996 (96.6%) survived to discharge, and 113 (11.0%) were admitted for a recurrence of AP. Inappropriate treatment had no impact on hospital stay (RRA 0.98, 95% CI 0.84–1.15) or hospital mortality (OR 1.02, 95% CI 0.47–2.19), although it was associated with a greater risk of admission because of recurrence (OR 3.7, 95% CI 2.4–5.8). **Conclusions**: We found that inadequate empirical treatment with cefazolin in adult patients with community-acquired acute pyelonephritis does not appear to change the length of hospital stay or in-hospital mortality in patients but is associated with an increased risk of readmission due to recurrence; this might favor the use of empirical narrow-spectrum antibiotics but with strategies that allow monitoring or early detection of microbiological non-eradication to prevent recurrence.

## 1. Introduction

Community-acquired acute pyelonephritis (AP) is a common infection in adults, especially in women of reproductive age, with a reported incidence rate of urinary tract infection of 0.5 to 0.7 per person-year. In young men aged 18–24, the incidence is 0.01 per person-year, while in postmenopausal women living in the community, it is 0.07 per person-year, and 0.12 per person-year in older women with diabetes [1]. In general, the disease has a good prognosis. However, risk factors such as age over 65 years and alterations in the immune or genitourinary systems may worsen its progression. The frequency of the disease is increasing, making it important to understand data related to its treatment [2,3]. In Colombia, a prevalence of urinary tract infection of up to 31% has been estimated in patients treated in the emergency room at a high-complexity center [4]. First-generation cephalosporins are a group of antimicrobials recommended for the treatment of acute pyelonephritis. The Colombian guidelines mention them as a first-line empirical treatment, and other Latin American guidelines, such as those from Argentina, also suggest their use as a valid option. Similarly, the National Institute for Health and Care Excellence (NICE) guidelines support their use [5,6,7]. However, there may be high rates of bacterial resistance to these medications; in Colombia, for example, a cefazolin susceptibility rate of 78.1% was reported in *Escherichia coli* isolates (the most significant etiological agent in UTIs) from patients with diabetes mellitus and acute pyelonephritis [8,9]. This has led to the empirical use of broader-spectrum antimicrobials, which, in turn, may contribute to increasing resistance. Therefore, it is essential to understand the impact of using narrow-spectrum antibiotics, such as cefazolin, in this infection [10].

The prevalence of community-acquired acute pyelonephritis caused by beta-lactamase-producing bacteria (ESBL) has increased in recent times, and varies by geographic location to more than 30% in Iran and some areas of China [11,12] to 10% in the United States of America [8,13] and 6.9% in Colombia [8]. Urinary tract infections caused by multidrug-resistant (MDR) bacteria have been associated with a longer length of hospital stay, with 6 (4–8) days compared to 5 (4–7) days for non-MDR bacteria (*p* = 0.029), so it is necessary to establish whether using inappropriate empirical treatment in the current situation of high resistance in acute pyelonephritis has any clinical impact [14].

The objective of this study was to evaluate the effect of inappropriate empirical therapy (which generally refers to administering the wrong treatment based on the in vitro susceptibility profile of the microorganism, including resistant and intermediate strains) with cefazolin on clinical outcomes, specifically hospital stay and recurrence, in adult patients with community-acquired acute pyelonephritis caused by resistant Enterobacterales requiring hospitalization in two hospitals in Colombia, where resistance rates have increased in recent years.

## 2. Results

Between January 2013 and January 2020, 12,460 patients with positive urine cultures were identified. Among them, 10.2% (1278) met the inclusion criteria, while 19.3% (247) met at least one exclusion criterion. Specifically, 25.1% (62) had incomplete data, 24.7% (61) had a urinary catheter, 23.5% (58) underwent antibiotic changes within the first 24 h, 12.5% (31) lacked an available susceptibility profile of the microorganism, 8% (20) had a history of previous urological surgery, 4% (10) had a diagnosis of hematological malignancy, and 2% (5) were infected with the human immunodeficiency virus.

**Figure 1:**
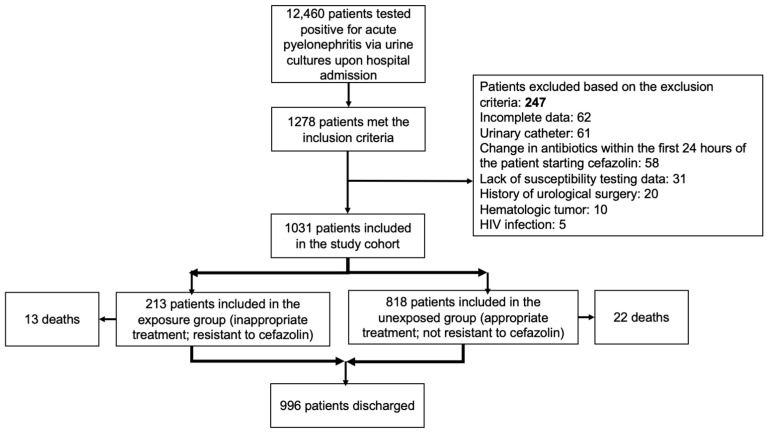
Patient flow chart.

Ultimately, 1031 patients were included in the definitive cohort. Among them, 21.1% (218) received inappropriate therapy due to cefazolin resistance (Figure 1). Patient characteristics were determined upon admission.

Among the included patients, 73.5% (758) were female, with an average age of 64.1 years (standard deviation (SD): 12.8 years). Among the women in the cohort, 68.1% (516) were postmenopausal, and 4.5% (34) were pregnant. The average Charlson Comorbidity Index score was 1.65 (SD: 2.16). The most common comorbidity was diabetes mellitus, present in 20.2% (208) of patients. Additionally, 71.6% (739) met at least one criterion for a complicated urinary tract infection (Table 1).

A total of 11.3% (117) of patients had received an antibiotic within 90 days before admission. Among them, 44.4% (52) had received a cephalosporin, 17.1% (20) a beta-lactam with an inhibitor, 11.1% (13) a quinolone, and 5.1% (6) a carbapenem. Other antimicrobials used included macrolides, clindamycin, nitrofurantoin, metronidazole, trimethoprim/sulfamethoxazole, fosfomycin, and tetracyclines.

### 2.1. Identified Microorganisms and Susceptibility to Cefazolin

Among the 1031 urine cultures from patients included in the present study, the most common microorganism was *E. coli*, accounting for 80.7% (832) of the isolates, of which 15.9% (133/832) were cefazolin-resistant; this was followed by *K. pneumoniae*, accounting for 7.3% (75), of which 33.3% (25/75) were cefazolin-resistant isolates, and *P. mirabilis*, accounting for 6.5% (67), of which 26.8% (18/67) were cefazolin-resistant. Four percent (42) of the Enterobacterales that produce chromosomal AmpC-type beta-lactamases were found to have resistance to cefazolin (conditioned by the chromosomal production of beta-lactamases): *M. morganii*, 1.7% (17); *Citrobacter* spp., 1.3% (12); *Klebsiella aerogenes*, 0.9% (9); *Serratia marcescens*, 0.3% (3); and *Proteus vulgaris*, 0.1% (1). Other Enterobacterales were identified in 1.3% (14) of the urine cultures and exhibited no resistance to cefazolin.

### 2.2. Outcomes in the Global Cohort According to the Type of Treatment

The mean length of hospital stay was 4.8 days (SD: 5.1). Before the propensity analysis, when stratified by treatment type, patients who received inappropriate treatment had a longer hospital stay, averaging 6.0 days (SD: 7.6), compared to 4.5 days (SD: 4.1) for those who received appropriate treatment (*p* < 0.001).

The mean duration of antibiotic administration across all patients was 9.5 days (SD: 3.5). For those who received inappropriate treatment, the duration was 9.37 days (SD: 3.5), while for appropriate treatment, it was slightly longer at 10.06 days (SD: 3.7) (*p* = 0.193).

A total of 10.9% of patients (113/1031) were readmitted for acute pyelonephritis (AP) within 30 days of discharge. Before the propensity analysis, a higher percentage of readmissions was observed in the inappropriate-treatment group, with 22.9% (50/218) compared to 7.7% (63/813) in the appropriate-treatment group (*p* < 0.001). The median time to readmission was 14.9 days (SD: 7.8), with no significant difference between exposure groups: 15.2 days (SD: 9.3) in the inappropriate-treatment group and 14.7 days (SD: 7.0) in the appropriate-treatment group (*p* = 0.664). Among patients who experienced recurrence, 36 urine cultures were performed: 12 in the inappropriate-therapy group and 24 in the appropriate-treatment group. The same microorganism from the initial AP episode was documented in 15 patients receiving appropriate therapy and in 6 patients from the inappropriate-therapy group.

The overall readmission rate for all causes was 21.7% (224/1031). A significantly higher proportion of readmissions was observed in the inappropriate-treatment group (34.9%, 76/218) compared to the appropriate-treatment group (18.2%, 148/813) (*p* < 0.001). Among the 1031 patients, 2.2% (23) required ICU admission. Before the propensity analysis, ICU admission was more frequent in the inappropriate-treatment group (5.0%, 11/218) compared to the appropriate-treatment group (1.5%, 12/813) (*p* = 0.003). Bacteremia secondary to acute pyelonephritis (AP) was observed in 2.5% (26) of patients, with no significant difference between treatment groups: 2.5% (20/813) in the appropriate-therapy group and 2.8% (6/218) in the inappropriate-therapy group. The antibiotic regimen was changed after 24 h in 25.2% (259/1031) of patients. A higher frequency of bacteremia secondary to AP was found among patients who received inappropriate treatment (46.5%, 99/218) compared to those who received appropriate treatment (19.6%, 160/813) (*p* < 0.001). Before inverse probability weighting (IPTW), 3.4% (35/1031) of patients died within 30 days, with a significantly higher mortality rate in the inappropriate-treatment group (6.1%, 13/218) compared to the appropriate-treatment group (2.7%, 22/813) (*p* = 0.025). Antibiotic therapy was modified in 259 patients (25.1%). Changes after 24 h were more frequent among those with resistant (103 of 218 cases, 46.3%) or intermediate isolates (81 of 203 cases, 39.5%) compared to patients with susceptible isolates (77 of 610 cases, 12.6%; *p* < 0.001 for all comparisons). The most common alternative antibiotics used were ceftriaxone (91 cases, 35.1% of changes) and carbapenems (85 cases, 32.8% of changes). Notably, carbapenem use was significantly higher in patients with cefazolin-resistant isolates compared to those with susceptible isolates (26.2% vs. 2.8%; *p* < 0.001).

### 2.3. Effect of Inappropriate Treatment on Clinical Outcomes

A multivariate count regression model (negative binomial) revealed no increase in the number of days to discharge in the group with inappropriate therapy, with an adjusted incidence rate ratio (aIR) of 0.98 (95% CI 0.84–1.15). This model also included the antibiotic change as an additional independent variable, which had an RTIa of 1.8, with a 95% CI of 1.58–2.06. Inappropriate therapy was associated with increased risks of overall readmission (OR 2.6, 95% CI 95% CI 1.8–3.7) and recurrence (readmission for pyelonephritis) (OR 3.7, 95% CI 95% CI 2.4–5.8). Using pseudopopulation, an excess of readmissions in the pseudopopulation of 3.9% and an excess of readmissions due to an AP of 3.4% were observed. Inappropriate therapy did not impact in-hospital mortality (OR 1.02 95% CI 95% CI 0.47–2.19). Table 2 shows the associations between inappropriate treatment and the outcomes of interest (with IPTW).

### 2.4. Sensitivity Analysis

The group of patients with nonsusceptible bacteria (that is, those identified as resistant or intermediate in the in vitro test) were evaluated for exposure, and 40.4% (416) were found to be exposed (resistant or intermediate) and 59.6% (615) were not exposed (susceptible). The proportion of ICU admissions was 3.4% (14/416) in this exposure group and 1.5% (9/615) in the nonexposure group. Of the patients with community-acquired acute pyelonephritis ultimately included for further susceptibility analysis, bacteremia was detected in 2.6% (11/416) of patients in the exposed group (those who received cefazolin and had a resistant or intermediate susceptibility profile) and in 2.4% (15/615) of the unexposed group, who had isolates with cefazolin susceptibility.

When performing the weighting adjustment (based on the propensity score), it was found that the use of inappropriate empirical antimicrobials did not affect the length of hospital stay or in-hospital mortality, with the same trend in the initial analysis. They were also found to have an increased risk of overall readmission and readmission for pyelonephritis. Using the pseudopopulation, an excess of readmissions for pyelonephritis of 8.0% and readmissions for AP of 5.7% were found. See Table 3.

In the subgroup analysis, 254 patients with a Charlson score of 3 or higher were selected. Among them, 21 (9.2%) died in the hospital, compared to a mortality rate of 1.8% in patients with a lower Charlson score (*p* < 0.001). In the adjusted proportional hazards Cox model, cefazolin resistance was not associated with mortality (aHR 1.1, 95% CI 0.42–2.8).

## 3. Discussion

Our study found that the empirical use of cefazolin as an inappropriate treatment in patients with community-acquired acute pyelonephritis (AP) due to Enterobacterales was not associated with prolonged hospital stay or mortality but was linked to an increased risk of general readmission and recurrence. Cefazolin is a widely used antibiotic for urinary tract infections (UTIs), yet data on its empirical use and the impact of resistance remain limited. This study provides valuable insight into the consequences of this approach. Although readmission for pyelonephritis was observed in a small number of patients, our findings raise questions about the best management strategies in cases of inappropriate therapy. The lack of an early clinical cure, as reflected in hospital stay duration, aligns with previous observations in patients with community-acquired febrile UTIs receiving appropriate treatment [15]. Other studies have reported different findings. For instance, in adult patients with *E. coli*-related UTIs in China, where 75.5% of cases were community-acquired, the use of inappropriate empirical antibiotics was associated with a longer hospital stay compared to appropriate treatment (13.6 ± 8.6 days vs. 10.8 ± 7.9 days, *p* = 0.008) [16]. Furthermore, in cases of UTIs caused by *K. pneumoniae*, a study found that 38% of infections were community-acquired, with 73.2% classified as complicated. In these patients, the use of inappropriate empirical treatment was associated with a higher risk of persistent urinary symptoms after 21 days and the need for a prolonged hospital stay [17].

Our study revealed an increased risk of recurrence. However, some authors have reported similar readmission and recurrence rates between patients receiving inappropriate and appropriate empiric treatment in AP. In the case of UTIs in adults caused by *E. coli*, the frequency of recurrence at 90 days with the use of inappropriate antibiotics was 3.9%, whereas it was 9.1% with the use of appropriate antibiotics (*p* = 0.125) [15]. Another study involving 30–60 days of follow-up in adult patients with AP who underwent inappropriate versus appropriate treatment reported a recurrence rate of 18% compared to 14%, respectively, without significant differences [18]. An investigation of urinary infection caused by ESBL-producing Enterobacterales in which appropriate antimicrobial agents were used was associated with higher recurrence rates at 30 days compared with that resulting from the administration of inappropriate treatment (42% versus 5%; *p* = 0.0003) [15]. These studies show contradictory results concerning the outcome of the recurrence of inappropriate empirical treatment, which could be solved by conducting randomized clinical trials.

The increasing antibiotic resistance of microorganisms involved in urinary tract infections is a global public health problem, so it is essential to develop strategies to mitigate this problem. One strategy may be the decision to shorten treatment durations, for example, to less than 7 days, which seems to offer a promising approach. This has been validated in both adults and children with urinary tract infections, including cystitis and acute pyelonephritis [19,20,21]. However, further research is needed to compare short-term therapy with long-term therapy, which emphasizes a duration of less than 7 days in pyelonephritis, because current evidence has emphasized comparisons between 7–10 days and more than 10–14 days [22]. Regarding recurrence with inappropriate treatment, this study opens the door for prospective studies that, for example, evaluate whether taking a control urine culture after inappropriate treatment could reduce this risk, since ensuring a microbiological cure along with clinical results is essential for curing the patient and avoiding recurrence [23].

The increased risk of readmission despite clinical improvement is likely due to microbiological persistence. A secondary analysis of 13 randomized clinical trials (RCTs) of patients with complicated UTIs submitted to the FDA reported that the discordance between clinical and microbiological outcomes was as high as 18%. Moreover, microbiological persistence in these patients was associated with a higher rate of late clinical relapse [24]. This finding is consistent with initial data on cefazolin use. A systematic review comparing first-generation cephalosporins in acute pyelonephritis with other antimicrobial agents, which included seven randomized clinical trials conducted before 2000, revealed no statistically significant differences in terms of clinical cure, length of hospital stay, or reinfection. However, it reported a lower probability of microbiological cure and a higher probability of relapse in the first-generation cephalosporin group, with no serious adverse effects observed [25].

A recent study compared cefazolin (7.1% resistance, 5/70) with ceftriaxone (4.9% resistance, 3/61) and revealed that cefazolin was noninferior to ceftriaxone with respect to clinical response, with no differences in length of hospitalization or readmission for cystitis or pyelonephritis after 30 days [26]. Together with our data, the use of cefazolin is a safe and effective first-line alternative that does not require longer hospital stays, as shown by several consensuses and guidelines [5,6,7]. Additional readmissions for pyelonephritis were observed in 3.4% to 5.7% of patients, depending on the definition used (resistant or intermediate). This finding highlights the potential need for follow-up urine cultures to guide therapy. For patients who receive inappropriate treatment but show favorable clinical progression, a follow-up urine culture 15 days after completing treatment could help confirm microbiological eradication and potentially prevent readmission. However, this approach remains controversial, as several studies have reported a high persistence of bacteriuria even after appropriate treatment. More clinical studies are needed to assess the impact of this strategy and determine its effectiveness in preventing recurrence [27,28]. Another potential approach would be to extend the duration of antibiotic therapy in cases of inappropriate treatment. However, further research is needed to determine the effectiveness and safety of this strategy. Additionally, in cases requiring extended treatment, performing susceptibility testing for oral antibiotics such as cephalexin would be ideal to ensure appropriate therapeutic selection. The U.S. Food and Drug Administration (FDA) also recommends assessing both microbiological and clinical cures when evaluating treatment success in urinary tract infection clinical trials [29]. To date, the best approach for patients with microbiological failure is unclear, given that this scenario has not been explored in clinical trials.

A similar pattern of microbiological failure to that observed with cefazolin was reported in a retrospective study on fluoroquinolones for UTIs caused by Enterobacterales. The study found that infection recurrence was associated with the minimum inhibitory concentration (MIC) value, with higher MICs leading to increased recurrence at 28 days (20.5% for low MICs, 25.3% for intermediate MICs, and 60% for high MICs; *p* < 0.01) [30]. Therefore, further research is needed to establish the correlation between antimicrobial susceptibility breakpoints and recurrence outcomes.

Our study found no impact of treatment type on in-hospital mortality, aligning with the RESCUING group’s findings on complicated UTIs. Inappropriate treatment was not linked to increased mortality risk; instead, mortality was associated with ICU admission, septic shock, and corticosteroid treatment [31]. In our study, the mortality rate (2.3%) was consistent with previous reports. This may be attributed to effective bacterial inoculum control in the urinary system due to the appropriate antibiotic concentration, such as cefazolin [32]. However, the mortality rate in people over 60 years of age, with a peak between 65 and 75 years, is 3.3/1000 people [33]. Age is a key factor associated with mortality in acute pyelonephritis and has been incorporated into mortality prediction scales for this condition, often using a cutoff of 65 years [34].

We found that a high percentage of recurrence cases were caused by the same microorganism as the initial infection. This finding is consistent with other research. For example, a study conducted at a university hospital in Jordan, which included 950 infection episodes in 650 patients, primarily urinary tract infections, reported that 65.1% of recurrent cases involved the same microorganism. Additionally, accurate empirical therapy, guided by the culture from the previous episode, was achieved in 71.8% of cases [23]. These findings highlight the importance of considering previous urine culture results in clinical practice to guide empirical antibiotic treatment for recurrent infections.

This study has several limitations, primarily its retrospective design. Although a large number of patients were included, antimicrobial selection, dosing, and treatment decisions were made by the treating physicians, resulting in a lack of standardized protocols for empirical cefazolin use. Additionally, the retrospective nature of the cohort introduces the possibility of information bias. Given that antimicrobial susceptibility patterns evolve over time, it is crucial to continuously update research to account for emerging bacterial resistance trends. However, a key strength of our study is the use of inverse probability weighting, which helped balance potential confounders by ensuring a distribution of covariates independent of exposure to inappropriate treatment.

## 4. Materials and Methods

### 4.1. Data and Subjects

We conducted a retrospective cohort study of patients with community-acquired acute pyelonephritis (AP) who were hospitalized for empirical antimicrobial management with cefazolin at two tertiary-level reference hospitals in Bogotá, Colombia: Clínica Universitaria (312 beds) and Hospital Universitario Nacional (230 beds). The study followed the STROBE methodology. Inclusion criteria encompassed adult patients (≥18 years) diagnosed with complicated or uncomplicated community-acquired AP who received cefazolin empirically (2 g intravenously every 8 h, adjusted for renal function if necessary) between January 2013 and January 2022. The diagnosis of AP was based on a positive urine culture and clinical findings indicative of systemic infection, sometimes supplemented by imaging studies. A complicated infection was defined as AP occurring in patients with comorbidities or structural/functional abnormalities of the urinary tract that increase the risk of complications, treatment failure, or recurrence. Examples include postmenopausal women, men, patients with urinary tract devices, and individuals with chronic kidney disease or diabetes mellitus [35]. An uncomplicated infection was defined as the absence of these risk factors. Exclusion criteria included the absence of antimicrobial susceptibility testing for cefazolin, lack of outcome data due to patient transfer within the first 48 h of admission, or antibiotic changes within the first 24 h of cefazolin initiation. Additional exclusions applied to patients with a permanent urinary catheter in place for over 30 days, lack of follow-up data post-discharge, a history of urological surgery, or a diagnosis of hematological malignancies or HIV infection.

Patients were identified through a microbiological information system (WHONET version 5.5, WHO) at the participating hospitals. The culture medium used was CHROMID CPS (BioMérieux, Marcy-l’Étoile, France), followed by isolation, identification, and susceptibility testing using the BioMérieux Vitek 2 system. Once colonies were isolated, MALDI-TOF was employed for precise identification of the etiologic agent. In cases of discrepancies, a second culture was processed, with the final identification based on MALDI-TOF results. Positive cultures were obtained for Enterobacterales, including *Escherichia coli*, *Klebsiella* spp., *Proteus* spp., *Enterobacter* spp., *Citrobacter* spp., *Serratia* spp., and other Enterobacterales. These microbiological findings were cross-verified with clinical medical records (Clinical Medical Record CMR, Sophia, version 2.18, and Hosvital) for hospitalized patients.

The diagnosis of acute pyelonephritis (AP) was made based on clinical and laboratory findings suggestive of the infection, as determined by the treating physicians. A positive urine culture for one of the identified pathogens confirmed the causal etiological agent. Data were collected using a structured clinical research format (CRF) specifically designed for the study and made available online through REDCap (Vanderbilt University, Nashville, TN, USA). To track readmissions, the Sophia and Hosvital systems were consulted, and hospitalization records were reviewed through the audit system of the patient’s insurer (Sanitas EPS, Bogotá, Colombia). Follow-up to assess readmissions was conducted for 30 days post-discharge.

### 4.2. Exposure

Exposure to cefazolin through the administration of antimicrobial agents in patients with isolates identified as nonsusceptible in the in vitro test was defined as an inappropriate therapy. The institutions’ clinical reference laboratory performed the urine culture with in vitro susceptibility testing via the broth dilution method with the automated Vitek system (BioMérieux, France). The methodology followed by the laboratory was recommended by the Clinical Laboratory Standards Institute (CLSI) [36].

### 4.3. Outcomes

Patients were included in the cohort when they had a diagnosis of community-acquired AP and were hospitalized for treatment. They were followed until the following outcomes were achieved: in-hospital mortality, hospital discharge, or readmission due to pyelonephritis to the same institution or another institution in the last 30 years.

The covariates collected were age (years), fever (temperature greater than 38.3 °C in the first 48 h of admission), and factors associated with complications (menopause, diabetes, physiological or anatomical alterations of the urinary tract, urolithiasis, or incontinence). Signs of a systemic inflammatory response (SIRS) were a temperature greater than 38 °C or less than 36 °C, heart rate greater than 90 beats per min, respiratory rate greater than 22 breaths per minute or CO_2_ blood pressure less than 32 mmHg, greater leukocyte count at 12,000 cells/mm^3^ or less than 4000 cells/mm^3^ [37], sepsis (based on the criteria of organ compromise derived from the infection, SEPSIS 3) [38], Charlson score, bacteremia (positive blood culture for the same microorganism identified in urine, taken within the first 72 h of admission), use of previous antibiotics (3 months), previous urological procedure (3 months), change in antibiotic (after 24 h of starting cefazolin), duration of hospitalization (time between admission and discharge), readmission (for any reason within 30 days of discharge), recurrence (readmission due to AP within 30 days of discharge), coincident urine culture (urine culture with the same microorganism identified in the first urine culture), admission to the intensive care unit (ICU, any admission during hospitalization), and death (death during hospitalization).

In the statistical analysis, to calculate the sample size, the following assumptions were used: resistance to cefazolin of 40%; duration of hospitalization, which could be extended from 4 days on average to 7 days [39]; and recurrence rate. In patients with antimicrobial resistance to quinolones, recurrence was observed in 10% of patients, whereas it occurred in approximately 4% of those who did not have antimicrobial resistance [40]. A total sample size of 840 patients (336 with inappropriate therapy and 504 with appropriate therapy) was determined to identify the difference in the expected recurrence rate, which served as the basis for performing the analysis according to the weighted inverse probability of inappropriate treatment.

The variables were described according to their distribution. Variables following a normal distribution are presented as means with standard deviations (SDs), while those with a non-normal distribution are reported as medians with interquartile ranges (IQRs). Categorical variables are expressed as percentages. To balance baseline characteristics between groups, a propensity score was used to adjust for potential confounders. A logistic regression model was constructed, with resistance as the outcome variable. The analysis was further refined using inverse probability weighting (IPW) based on the propensity score. In this approach, exposed individuals (those receiving inappropriate therapy) were assigned a weight of 1/propensity score × prevalence of exposure, while unexposed individuals were assigned a weight of 1/(1 − propensity score) × prevalence of nonexposure. This weighting method helped achieve comparability between groups by balancing covariates independently of treatment exposure.

From the weighting, a pseudopopulation was obtained for analyzing hospitalization time via a counting model, where the outcome variable (dependent) was the time to discharge and the predictor variable was inappropriate therapy (resistance).

An adjustment was made for possible covariates (independent) that occurred after admission, such as adjustment for antimicrobial treatment, changes in oral treatment, and admission to the ICU. The model assumptions and overdispersion were evaluated, and various approximations were carried out with different distributions (Poisson, exponential, negative binomial) both graphically and by error evaluation (using the AIC). The model whose distribution had the smallest error was chosen. Robust errors were used for the confidence interval calculation of the adjusted incidence rate ratios.

For the outcomes of readmission, recurrence, and in-hospital mortality, logistic regression models were used with resistance as a predictor variable. Adjustments were made for antimicrobial treatment (which could have been modified after admission) and other variables not included in the propensity score that arose post-admission. Robust standard errors were applied to calculate confidence intervals for the adjusted odds ratios (ORs). All statistical analyses were conducted using the R statistical software (R Foundation, Vienna, Austria), version 1.31056, with the appropriate libraries. A sensitivity analysis was performed to assess the impact of inappropriate therapy. The same statistical methods were applied using inverse probability weighting (IPW) for exposure adjustment. Additionally, a second definition of exposure was considered, including patients with intermediate in vitro susceptibility. The effects of this broader definition of inappropriate therapy were evaluated for the same outcomes: length of hospital stay, all-cause readmission, pyelonephritis-related recurrence, and in-hospital mortality.

A subgroup analysis was conducted on patients with a Charlson score greater than 2 to assess the impact of resistance on mortality. Specifically, patients with a Charlson score of 3 or more were selected, and a propensity score was calculated. An inverse probability of treatment weighting (IPTW) approach was applied to control for confounding. Multivariable Cox proportional hazards regression models were then used to evaluate in-hospital mortality.

## 5. Conclusions

Our study found that inappropriate cefazolin treatment in adult patients with community-acquired acute pyelonephritis does not impact hospital stay duration or in-hospital mortality but is associated with a higher risk of readmission and recurrence. Further prospective studies are needed to identify monitoring strategies for early detection of microbiological non-eradication, which could help prevent recurrence and support the empirical use of narrow-spectrum antibiotics.

## Figures and Tables

**Table 1:** Characteristics of the patients included in the study before and after weighting adjustment (based on the propensity score).

Variable	Before Weighting Adjustment				After Weighting Adjustment		
	Appropriate Treatment (Not Exposed) (N = 813)	Inappropriate Treatment (Exposed) (N = 218)	DME ^&^	*p*	Appropriate Treatment	Inappropriate Treatment	DME ^&^
Male sex n (%)	206 (25.3)	67 (30.7)	0.120	0.129	218.5 (26.8)	57.8 (26.7)	0.003
Age years (SD) *	62.68 (22.28)	69.27 (19.21)	0.317	<0.001	63.98 (21.89)	63.37 (21.52)	0.028
Diabetes mellitus n (%)	167 (20.5)	39 (18.8)	0.044	0.637	21.1 (2.6)	8.0 (3.7)	0.063
Menopause n (%)	397 (48.5)	119 (55.9)	0.139	0.082	404.1 (49.6)	103.5 (47.8)	0.036
Pregnancy n (%)	30 (3.7)	4 (1.9)	0.113	0.251	27.9 (3.4)	7.6 (3.5)	0.004
Charlson Index (SD)	1.52 (2.04)	2.15 (2.51)	0.273	<0.001	1.64 (2.14)	1.57 (2.15)	0.033
Previous antibiotic use n (%)	87 (10.6)	30 (14.1)	0.105	0.252	91.9 (11.3)	24.6 (11.4)	0.003
General discomfort n (%)	342 (41.8)	87 (40.8)	0.020	0.851	339.2 (41.6)	84.9 (39.2)	0.048
Fever n (%)	327 (40.0)	78 (36.6)	0.069	0.338	318.6 (39.1)	90.7 (41.9)	0.058
Asthenia n (%)	258 (31.5)	77 (36.2)	0.098	0.278	263.5 (32.3)	67.2 (31.1)	0.027
Low back pain n (%)	243 (29.7)	53 (24.9)	0.108	0.126	228.6 (28.0)	74.6 (34.4)	0.138
Temperature °C average (SD)	36.94 (0.93)	36.89 (0.84)	0.055	0.355	36.93 (0.92)	36.98 (0.88)	0.058
Average (SD) BPM ^+^ heart rate	90.44 (17.76)	88.36 (17.64)	0.117	0.053	90.08 (17.78)	90.30 (17.73)	0.012
Average respiratory rate RPM ^++^ (SD)	18.74 (2.25)	18.59 (2.02)	0.070	0.355	18.71 (2.23)	18.70 (1.92)	0.004
SIRS ^+++^ n (%)	241 (29.5)	50 (23.5)	0.005	0.122	231.6 (28.4)	61.9 (28.6)	0.005
Sepsis n (%)	174 (22.8)	65 (32.3)	0.023	0.234	190.9 (23.4)	48.6 (22.4)	0.023
CRP ^-^ mg/dL average (SD)	73.93 (89.77)	70.23 (82.76)	0.043	0.512	73.99 (90.16)	69.25 (82.29)	0.055
Creatinine mg/dL average (SD)	1.09 (0.72)	1.32 (1.04)	0.257	<0.001	1.15 (0.84)	1.16 (0.81	0.014

* SD: standard deviation; ^+^ BPM: beats per minute; ^++^ RPM: respirations per minute; ^+++^ SIRS: signs of systemic inflammatory response; ^-^ CRP: C-reactive protein; ^&^ DME: standardized mean difference.

**Table 2:** Impact of inappropriate treatment (cefazolin resistance) on adjusted outcomes (with IPTW).

	Association Measure	Worth	Confidence Interval ***	
			Upper Limit	Lower Limit
Hospital stay	RTIa *	0.98	0.84	1.15
Re-entry	OR **	2.63	1.83	3.79
Recurrence	OR **	3.76	2.43	5.81
In-hospital mortality	OR **	1.02	0.47	2.19

RTIa *: adjusted incidence rate ratio; OR **: odds ratio. *** Robust 95% confidence interval.

**Table 3:** Impact of inappropriate treatment (cefazolin resistance and intermediate) on adjusted outcomes (with IPTW).

	Association Measure	Worth	Confidence Interval ***	
			Upper Limit	Lower Limit
Hospital stay	RTIa *	0.91	0.79	1.05
Re-entry	OR **	3.08	2.25	4.22
Recurrence	OR **	3.79	2.46	5.84
In-hospital mortality	OR **	0.72	0.35	1.46

RTIa *: adjusted incidence rate ratio; OR **: odds ratio. *** Robust 95% confidence interval.

## Data Availability

The original contributions presented in this study are included in the article. Further inquiries can be directed to the corresponding author.
